# P02-03 A systematic review and qualitative meta-synthesis of the context, barriers and facilitators to cross-sector collaboration promoting physical activity

**DOI:** 10.1093/eurpub/ckac095.022

**Published:** 2022-08-29

**Authors:** Vasiliki Kolovou, Nicola Bolton, Diane Crone

**Affiliations:** School of Sport & Health Sciences, Cardiff Metropolitan University, Cardiff, United Kingdom

**Keywords:** systems thinking, cross-sector collaboration, public health interventions, health promotion, community sport

## Abstract

**Background:**

The intricacies of cross sector partnerships regarding what may help and what may hinder collaboration and partnerships in the short- or long-term remain largely unknown. The 2018 Global Action Plan for Physical Activity promotes whole-system collaborative approaches and our review contributes to our understanding of the management of cross-sector partnerships and collaborations promoting physical activity.

**Methods:**

We searched Medline, Embase, PsychINFO, ProQuest Central, SCOPUS and SPORTDiscus to identify published records dating from 1986 to August 2021. Public health interventions using a partnership approach were included, if at least two partners were not from the same sector and their shared goal was to promote or increase population-level physical activity. Eligibility also required records to include information about the context and the barriers and/or facilitators of the partners of the reported cross-sector partnerships. The CASP checklist and the ROBINS-I tool were used to appraise the included records and thematic analysis to summarise and synthesise the findings. Due to the small number of eligible quantitative studies, the study focused on qualitative findings relating to the parameters set out for this systematic review.

**Results:**

The study included records (n = 32) that described public health interventions or programs aiming to promote physical activity, community sport or active living through cross-sector partnerhips, as part of qualitative (n = 19), quantitative (n = 1) or mixed methods (n = 12) research. The review identified barriers, facilitators and recommendations to overcome common challenges in relation to four broad themes: approaching and selecting partners, funding, building capacity and taking joint action.

**Conclusion:**

Partners are often urged to collaborate under the limits of time and resources. Agreeing on expectations, gaining momentum and establishing trust requires time and resources early on, before any intervention output can be evidenced. Therefore, investment in the early stages may remove key barriers of partnerships and accelerate joint leadership. Boundary spanners linking sectors, translating differences and consolidating common ground can facilitate whole-systems approaches.

